# Identification of high-risk groups for complication after arthroplasty: predictive value of patient’s related risk factors

**DOI:** 10.1186/s13018-018-1036-2

**Published:** 2018-12-29

**Authors:** Martha Cecilia Castano-Betancourt, Ricardo Fruschein Annichino, Marcelo de Azevedo e Souza Munhoz, Eduardo Gomes Machado, Monica Vannucci Lipay, Evaldo Marchi

**Affiliations:** Laboratory of Genetics Epidemiology, Faculty of Medicine of Jundiaí (FMJ), Rua Francisco Telles 250, Vila Arens, Jundiaí, SP 13202-550 Brazil

**Keywords:** Arthroplasty, Complications, Prediction, Rheumatoid arthritis, Osteoarthritis

## Abstract

**Background:**

Total joint arthroplasty (TJA) benefit patients with osteoarthritis (OA) and rheumatoid arthritis (RA). However, a specific approach to detect patients at higher risk of prosthetic joint infection (PJI) and mechanical complications is absent. The aim of this study is to identify groups at higher risk for infections and mechanical complications after TJA in patients with RA and OA based on their most significant predictors.

**Methods:**

This is a hospital-based cohort study with 1150 recipients of TJA. Risk factors and comorbidities were assessed prior to the index surgery. Multivariate logistic and hazard regression were used to determine the relationship between risk factors and occurrence of complications after TJA. Odds ratios (OR), hazard ratios (HR), 95% confidence intervals (CI), and comparison between areas under the curve (AUC) using DeLong’s method are presented.

**Results:**

Complications were more frequent in subjects with RA, use of corticosteroids, and previous comorbidities: respiratory disease, infections, diabetes, anemia, mental and musculoskeletal comorbidities than in subjects without these risk factors, and these factors were predictors of infections and mechanical complications (*P* < 0.05). A model including these factors was superior to a model with only type of joint disease (OA/RA) or age and gender to detect infections or mechanical complications after TJA (*P* < 0.05 for difference between models). Complication risk proportionally increased with the presence of two or more comorbidities (*P* < 0.001).

**Conclusions:**

There are two groups at higher risk for infections after TJA: patients with OA with at least two risk factors and patients with RA, who usually present at least one of the risk factors for infection.

## Introduction

Total joint arthroplasty (TJA) is a successful operative intervention for subjects with osteoarthritis (OA), rheumatoid arthritis (RA), and other joint problems, reducing pain and increasing joint mobility. Each year, more than one million total knee and total hip replacements are performed in the USA, and these numbers are expected to rise worldwide due to aging population [1]. It is known that about 25% of subjects diagnosed with RA will require TJA and they will benefit from this surgery as well as patients with OA [2, 3]. However, subjects with RA have more complications after TJA than subjects with OA, especially prosthetic joint infection (PJI) and mechanical complications [4–7]. As it is known, data pertaining to PJI and mechanical complications in patients with RA and other inflammatory arthritis undergoing TJA is very limited [8].

Despite being less frequent than other type of complications, PJI is one of the most serious complications, leading to prolonged hospitalization, surgical revision, or even death [5, 6]. The incidence of PJI has been reported to be between 2 and 5% in developed countries, with an overall mortality of 3.4% to 15.2% within 5 years [5, 6]. Superficial surgical site infection, prior to joint arthroplasty or infection, advanced age, diabetes mellitus, and depression, among others, have been mentioned as risk factors for PJI [7–11]. RA and other immunocompromised conditions including use of corticosteroids have been pointed as risk factors for infections principally following total knee arthroplasty (TKA) [11–14]. However, awareness of the high risk that patients with RA and other associated risk factors have for complication after arthroplasty and their predictive value still need to be determined.

Most of the studies on risk factors for complications after TJA focused on patients with OA. There are a few studies on RA patients about risk factor for PJI or mechanical complications after TJA when compared with OA cases [12, 13]. Although from more than 10 years ago, RA has been considered as a risk factor for PJI; there is no evidence on risk factors or comorbidities that might contribute to explain the increased risk of complications in patients with RA [11, 15]. Therefore, in this study, we aim to identify the predictive value of RA and other factors that increase the risk for infections (superficial/PJI) and mechanical complications after TJA, allowing identification of high-risk groups. The identification of factors that predict which patients might suffer a complication after TJA will help in patient’s counseling before surgery and have an effect on surgical decision-making process, decreasing complications. It might also guide clinicians in preventing PJI in patients with RA undergoing TJA.

## Materials and methods

### Study population

**Fig. 1:**
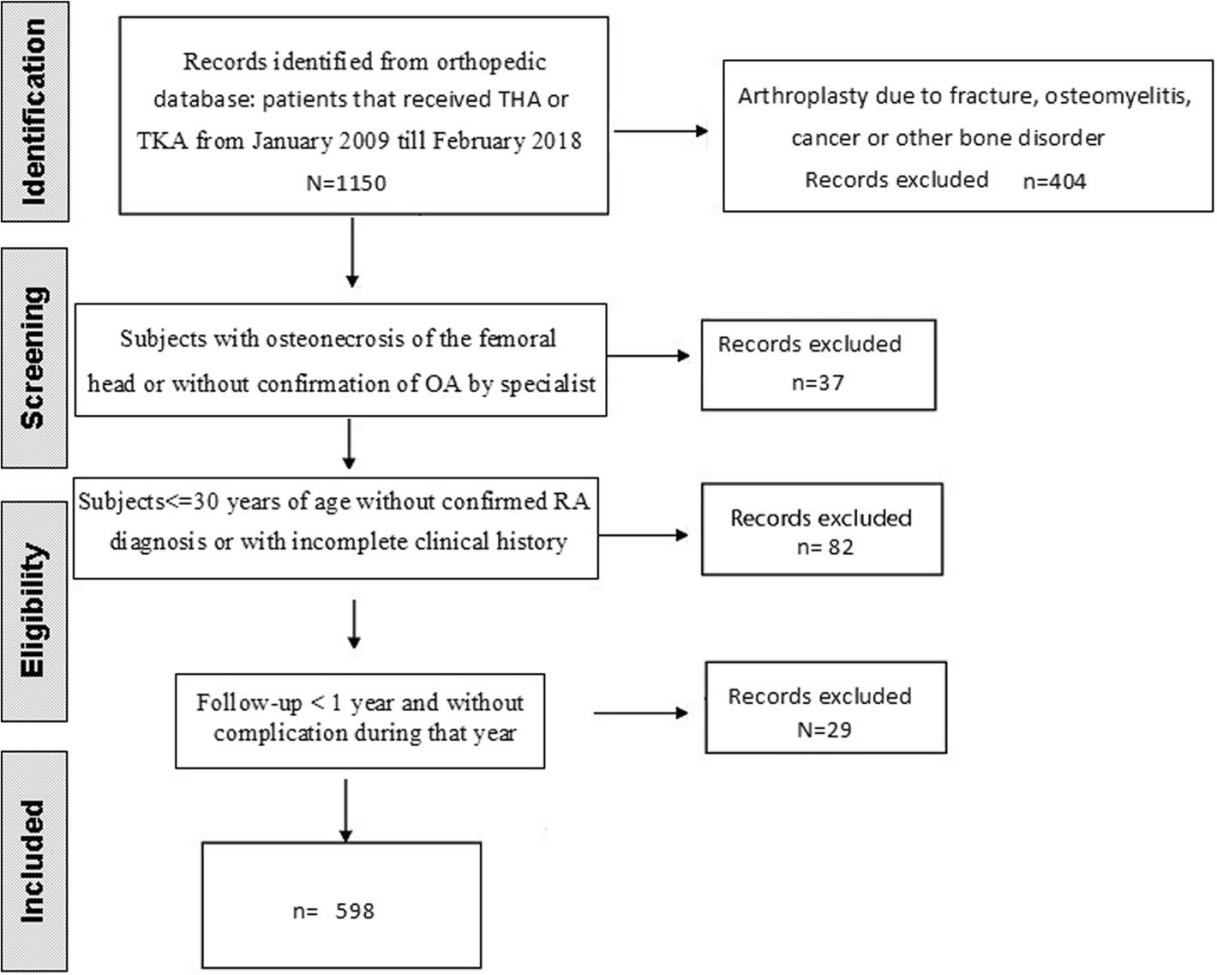
Flowchart of the study population

This is a retrospective hospital-based cohort study of men and women aged 30 years and older. The orthopedic department of the hospital has a consult-unit dedicated to subjects with hip and knee pathologies requiring arthroplasty or revision. We checked hospital records of subjects who in the previous years received a primary joint replacement. Majority of those identified subjects received a TJA between January 2009 and February 2018 and some before 2009. We identified records of 1150 patients that received a primary total hip or total knee arthroplasty (Fig. 1, flowchart). Exclusion criteria were based on all other reasons for arthroplasty not concerning to OA or RA. Subjects with TJA due to fractures, osteomyelitis, cancer, and other bone disorders were excluded (*n* = 404). In addition, patients with avascular necrosis or without confirmation of OA by specialist (37), patients 30 years old or younger with uncertain/not verified RA diagnosis or with incomplete clinical history (82), and patients with a follow-up of less than 1 year and without complication (29) were excluded. Electronical records and/or interview of the subjects included demographic information, anamnesis, diagnosis, date and joint of surgery, complication date and type, laboratory measurements, medication, smoking and alcohol history, previous hospitalizations, past surgeries, consults to the hospital (previous or within the studied period), and comorbidities. Protocol before surgery included pre-anesthetic consultation, laboratory exams (urea, creatinine, complete blood count, glycemia, coagulation tests, prothrombin time of activity), electrocardiogram, chest X-ray, prophylaxis for deep vein thrombosis (low-weight enoxaparin for 15 days), dental examination and intravenous antibiotic prophylaxis for 24 h. Radiographs of the joints: hips and/or knee and spine (each joint(s) were graded based on “The American College of Rheumatology” (ACR) and the “European League Against Rheumatism” (EULAR) or Kellgren and Lawrence score (KL). Diagnosis of definite RA and OA were made by an orthopedist and/or rheumatologist prior to surgery. Selected patients were interviewed at the hospital or by phone to complete missing data of hospital records. Subjects with severe pain and evidence of advanced joint destruction (KL ≥ 3) were placed in waiting list for surgery. For knee arthroplasty was used a cobalt-chrome cemented prosthesis. In all knee cases, were used models of prosthesis with posterior cruciate sacrifice. Hip arthroplasty was performed with a hybrid prosthesis (cemented stem and cementless cup) with capsular and external rotators repair with a posterior surgical approach (Moore’s approach).

Comorbidities were identified, classified, or converted to the ICD-10 codes. We only registered comorbidities when it occurred prior to the index surgery. Complications were identified through revision of clinical history and/or list of patients requiring revision. The type of complication was verified by the specialist. We registered all complications and causes of TJA revision from index surgery and within the study period. Complications were classified as mechanical (dislocation, osteolysis in prosthesis with less than 6 years old, loss of implant fixation, implant failure, fracture), infectious (superficial and/or deep), systemic events (thromboembolic events, cardiorespiratory arrest, hypovolemic shock or stroke in the first 90 days following surgery), and others (arthrofibrosis and legs length discrepancy after surgery of more than 2 cm). Time between surgery and complication was calculated in number of days/years.

Comorbidities listed on hospital discharge or by inter-consult request before the index surgery were classified as cardiovascular, cancer, infections (urinary tract infection and respiratory infection, between others), gastrointestinal, mental (anxiety, depression, bipolar disorder, schizophrenia, and other mood disorders), respiratory, musculoskeletal (osteoporosis, hernia, low back pain, spinal disc herniation, muscle tears, tendon calcification, carpal tunnel syndrome, hallux valgus, between others), and others (not classify in mentioned groups).

Univariate analysis was used to compare demographics of subjects with TKA or THA at the time of the index. Chi-square or Mantel-Haenszel statistics and Fisher’s exact tests were used to compare categorical variables. ANOVA (*F* test) was used to determine whether difference between groups was significant for continuous variables. Hazard regression and multivariate logistic regression models were used to estimate the association between binary risk factors and all type of complications, mechanical and complications due to infections in all subjects or in subjects with RA only (adjusted for potential confounders: age, sex).

Area under the receiver operating characteristic curve (AUROC) were calculated and compared for models containing only age and gender and models containing significant risk factors for mechanical complications and PJI after TJA. The ideal model would have an AUROC of 1, whereas a random guess would have an AUROC of 0.5. ROC analysis provides a useful means to assess the diagnostic accuracy of a model and to compare the performance of more than one variable for the same outcome. We used the DeLong’s method (1988) to compare AUROC between the models, where a *P* < 0.05 means that one model predicts better outcome than the other [16]. The probability of a type I error was set to 0.05 for all analyses. MedCalc for Windows (MedCalc Software, Ostend, Belgium), version 15.0 and SPSS 23.0 were used to perform the statistical analyses. The medical ethic committee of the University and National Commission for Research Ethics approved the study (06/10/2015), and therefore this study has been performed in accordance with the ethical standards laid down in the 1964 Declaration of Helsinki. Informed consent was obtained from all individual participants included in the study.

## Results

Between January 2009 and February 2018, 598 TJA were reported in the hospital records in patients with OA and RA. The electronical record system started in the hospital in January 2009. Most of the population consisted of workers of low income (90% with two or less minimum salaries per month). Patients with RA (8%) were younger, heavier, majority females, and with greater number of comorbidities (respiratory, infections, anemia, musculoskeletal, and other comorbidities) than patients with OA (see Table 1). Patients with RA used more corticosteroids. Prednisone was taken orally by 30%, leflunomide 4%, methotrexate 13%, and 39% were not under RA-treatment.

**Table 1:** Characteristics of the population receiving a total joint arthroplasty (TJA) according to diagnosis

Characteristics	Osteoarthritis (*n* = 552)	Rheumatoid Arthritis (*n* = 46)	*P* value
Age (years): mean (SE)	69 (0.4)	64.3 (1.8)	0.005
Sex (Female)	336 (58)	37 (79)	0.007
Diabetes	148 (27)	13 (28)	0.87
Hypertension	372 (69)	30 (65)	0.58
Osteoporosis^a^	104 (19)	13 (28)	0.12
Smoking	95 (18)	7 (15)	0.51
Alcohol	58 (11)	3 (7)	0.37
Corticosteroids use	22 (4)	17 (37)	< 0.001
Obesity (BMI > 30)^b^	102 (56)	18 (64)	0.62
Respiratory comorbidity	43 (8)	9 (20)	0.009
Infectious comorbidity	47 (9)	11 (24)	< 0.001
Anemia	11 (2)	7 (15)	< 0.001
Osteomuscular comorbidity	122 (22)	18 (39)	0.001
Mental comorbidity	44 (8)	1 (2)	0.15
Cardiovascular comorbidity	110 (20)	12 (26)	0.32
Other comorbidity	97 (18)	21 (46)	< 0.001
Gastrointestinal comorbidity	36 (7)	5 (11)	0.26
Cancer	25 (5)	0 (0)	N.A.
Type of surgery (THA)^c^	314 (57)	27 (59)	0.81
Complications (*n* = 98)	78 (13)	21 (45)	< 0.001
Complications by surgery (THA)	48 (62)	12 (57)	0.31

Values are numbers and in parenthesis () standard deviations for continuous values or percentage for categorical variables (%). All variables were adjusted by age and gender. *BMI* body mass index

^a^Subject diagnosed and/or receiving treatment for osteoporosis (from them, 87% had at least one previous fracture)

^b^BMI calculated from measures at personal interview

^c^*THA* total hip arthroplasty compared with *TKA* total knee arthroplasty

**Fig. 2:**
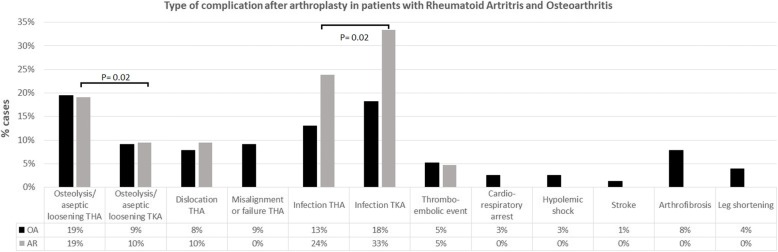
Type of complication after arthroplasty in patients with rheumatoid arthritis (RA) and osteoarthritis (OA). Total hip arthroplasty (THA), total knee arthroplasty (TKA). *P* value for difference in percentage (%) of complication between THA and TKA cases

Majority of the patients in that record (95%) received a TJA between 2009 and 2018. The follow-up period ranges from 1 day for those patients having a complication next day after surgery to 16 years for the oldest TJA (mean follow up = 4.96). During that period, 98 (16.6%) patients had complications and 48 of them had to undergo a revision surgery. Most of the complications were classified as mechanical and infections (*n* = 43 and *n* = 38, respectively). Mechanical complications were principally due to osteolysis, aseptic loosening of the implant (28), and dislocation of THA (*n* = 11) (Fig. 2), including a subject with both types of complications. Infections were superficial (29%) and PJI (71%). Systemic complications included thromboembolic events (5), cardiorespiratory arrest (2), hypovolemic shock (2), and stroke (1). Most systemic complications occurred in subjects with OA (Fig. 2), during the first three postoperative days. One (1) subject received blood transfusion and four (4) systemic complications were fatal. Other complications not classified in the mentioned groups were arthrofibrosis and length discrepancy of lower limbs > 2 cm (Fig. 2, nine cases). There were no significant differences in number of complications between THA and TKA (Table 1, *P* = 0.81). However, mechanical complications (osteolysis and dislocations) were 20% more common in THA (Fig. 2) and infections were more common in TKA cases (Table 2 and Fig. 2, *P* = 0.02 for both).

**Table 2:** Association of risk factors and comorbidities with all-type, mechanical, and complications due to infections after total joint arthroplasty (TJA)

	All type complications (*n* = 92)		Mechanical complication^a^ (*n* = 43)		Infection complication^b^ (*n* = 38)	
	HR (95%CI)	*P*	HR (95%CI)	*P*	HR (95%CI)	*P*
Risk factors						
Age	1.00 (0.98–1.02)	0.99	0.99 (0.97–1.02)	0.49	1.00 (0.97–1.03)	0.98
Gender (female)	0.94 (0.63–1.40)	0.75	0.85 (0.46–1.57)	0.61	1.30 (0.65–2.60)	0.46
Smoking	1.29 (0.85–1.97)	0.23	1.14 (0.51–2.56)	0.75	1.64 (0.72–3.74)	0.24
Alcohol	1.22 (0.74–2.02)	0.44	1.39 (0.54–3.55)	0.50	0.84 (0.25–2.85)	0.78
Corticosteroids	*2*.*14* (*1*.*16*–*3*.*92*)	*0*.*01*	1.68 (0.60–4.75)	0.33	*3*.*16* (*1*.*39*–*7*.*22*)	*0*.*006*
Comorbidities						
Diabetes	1.42 (0.93–2.15)	0.10	1.10 (0.56–2.16)	0.79	*2*.*32* (*1*.*18*–*4*.*57*)	*0*.*02*
Obesity	1.15 (0.56–2.34)	0.70	1.72 (0.87–3.39)	0.12	1.58 (0.72–3.47)	0.26
Respiratory	*2*.*35* (*1*.*39*–*3*.*97*)	*0*.*001*	0.94 (0.29–3.12)	0.94	*4*.*55* (*2*.*03*–*10*.*2*)	*< 0*.*001*
Anemia	*2*.*46* (*1*.*07*–*5*.*70*)	*0*.*04*	NA	NA	4.93 (1.90–12.8)	0.001
Osteoporosis	1.34 (0.85–2.12)	0.21	1.83 (0.94–3.56)	0.08	1.08 (0.50–2.35)	0.84
Infectious	*1*.*91* (*1*.*13*–*3*.*23*)	*0*.*02*	1.51 (0.64–3.60)	0.35	*3*.*09* (*1*.*50*–*6*.*40*)	*0*.*002*
Musculoskeletal	1.42 (0.92–2.18)	0.12	*2*.*11* (*1*.*13*–*3*.*96*)	*0*.*02*	1.15 (0.56–2.37)	0.71
Hernia (all-type)	1.84 (0.98–3.44)	0.06	*3*.*93* (*1*.*77*–*8*.*72*)	*0*.*001*	NA	NA
Mental	*2*.*16* (*1*.*24*–*3*.*75*)	0.03	1.14 (0.35–3.72)	0.83	*2*.*50* (*1*.*09*–*5*.*70*)	*0*.*05*
Gastrointestinal	1.12 (0.52–2.42)	0.78	1.18 (0.36–3.84)	0.79	0.87 (0.27–2.83)	0.82
Cancer	0.74 (0.24–2.35)	0.62	0.59 (0.08–4.30)	0.60	1.33 (0.32–5.60)	0.70
Cardiovascular	1.12 (0.69–1.80)	0.65	0.90 (0.41–1.97)	0.79	1.02 (0.47–2.24)	0.96
Others	*1*.*89* (*1*.*23*–*3*.*90*)	*0*.*004*	1.99 (1.03–3.85)	0.04	1.65 (0.82–3.34)	0.16
TKA	0.93 (0.62–1.40)	0.73	*0*.*41* (*0*.*19*–*0*.*87*)	*0*.*02*	*2*.*18* (*1*.*11*–*4*.*29*)	*0*.*02*
RA	*3*.*81* (*2*.*30*–*6*.*31*)	*< 0*.*001*	*3*.*25* (*1*.*46*–*7*.*25*)	*0*.*004*	*5*.*98* (*2*.*93*–*12*.*2*)	*< 0*.*001*

*TKA* total knee arthroplasty (compared to THA), *RA* rheumatoid arthritis. All variables were adjusted for age/gender, any kind of infection, including urinary tract and respiratory infections

^a^Mechanical complication: dislocation, osteolysis in prosthesis with less than 6 years old, loss of implant fixation, implant failure, fracture

^b^Infection-complication: superficial and deep infections, called prosthetic joint infections (PJI). In italic all significant factors (*P* value < 0.05)

Risk factors for all type of complications were RA, respiratory comorbidity, anemia, any previous infection (majority urinary tract infection and respiratory infection), use of corticosteroids, and other comorbidities (Table 2, *P* ≤ 0.05). Risk factors for mechanical complications were RA and history of a previous musculoskeletal comorbidity, principally osteoporosis (35% of cases with mechanical complication), and hernia (19% of cases with mechanical complication) as the most relevant factors (Table 2, *P* ≤ 0.05). Osteoporosis was principally associated with cases of osteolysis or periprosthetic fractures (OR 2.5, CI 1.26–4.84) and hernia with dislocation (OR 5.4, CI 1.3–22).

**Fig. 3:**
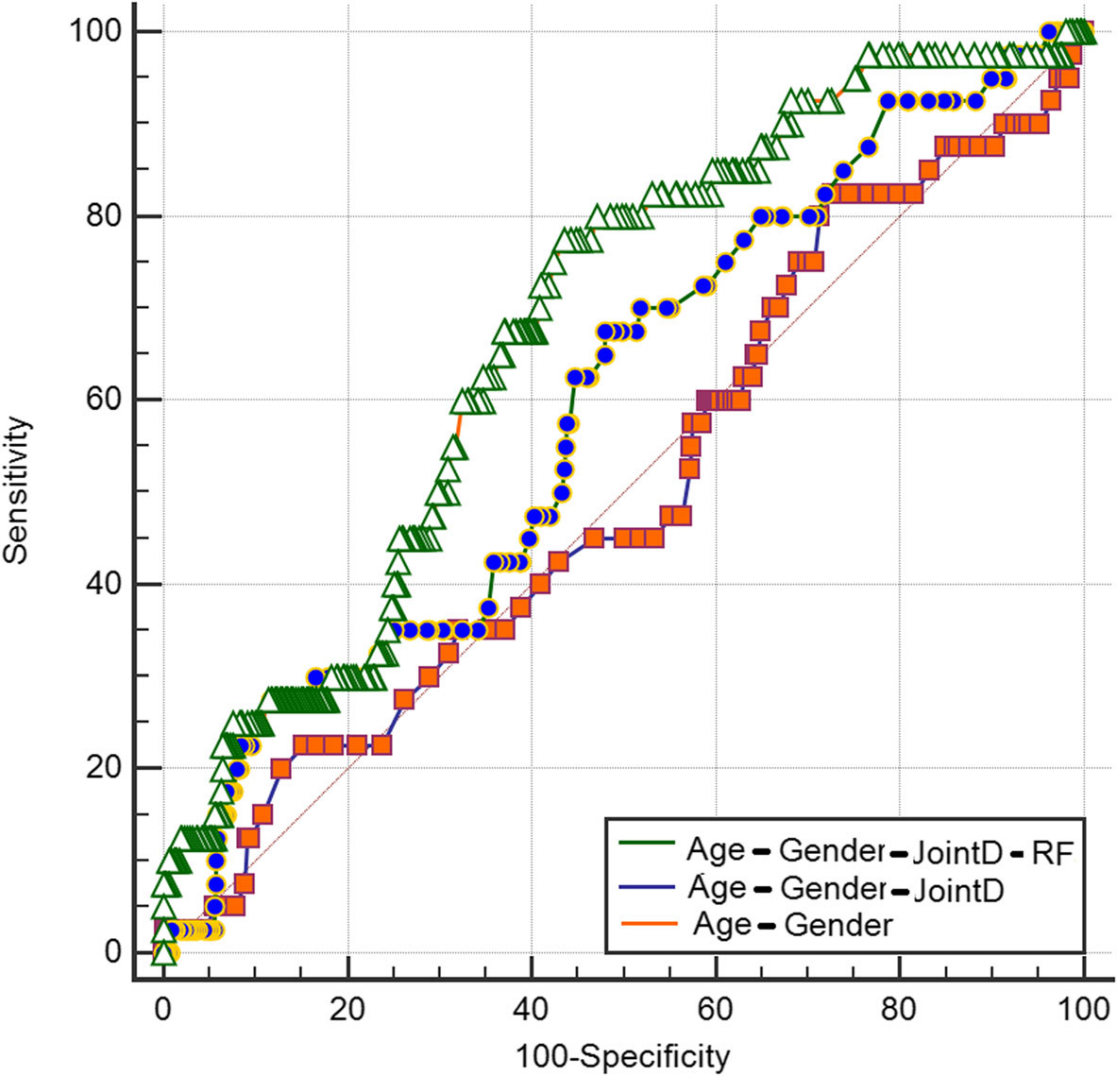
Comparison of areas under the receiver operating characteristic curve (AUROC) for mechanical complications. Fist model (orange line) included only age and gender of patients, second model (blue line): age, gender, and type of joint disease (JointD): osteoarthritis or rheumatoid arthritis. Last model (green line); all these factors plus history of musculoskeletal comorbidity. Difference between AUROC was only significant for model 3 compared with model 1

Regarding the predictive value of these factors, a model including type of joint disease (RA or OA) and history of any other musculoskeletal comorbidity increased the prediction of cases with mechanical complications compared with a model with only age and gender (Fig. 3, AUC = 0.68 and 0.51 respectively, *P* = 0.003 for difference between AUROCs). The contribution of type of joint disease (RA/OA) was not significant in the prediction of mechanical complications compared with a model containing only age and gender (*P* = 0.26 for difference between AUROCs).

**Fig. 4:**
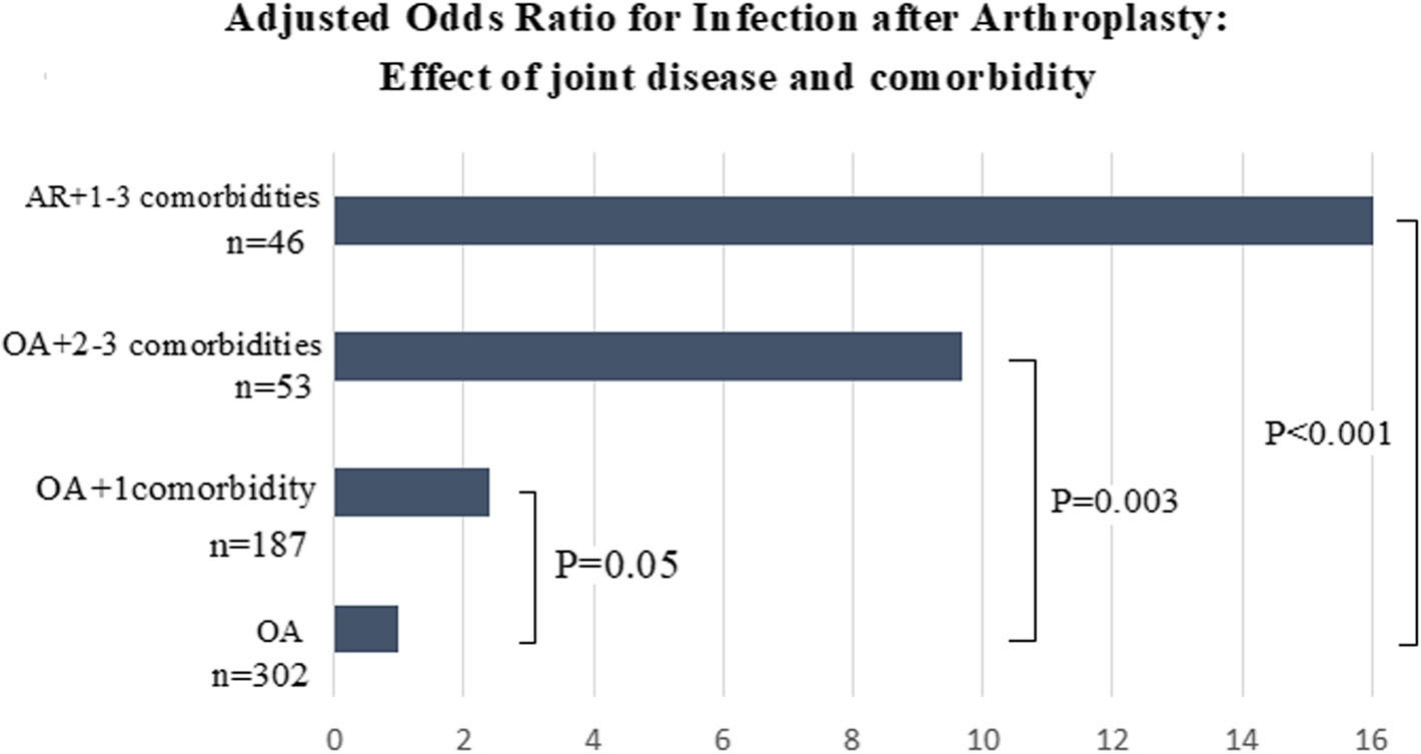
Adjusted odds ratio for complication due to infection after arthroplasty: effect of joint disease and comorbidities. Group 1 (reference): patients with osteoarthritis (OA) without risk factors for infection. Rheumatoid arthritis (RA)

**Fig. 5:**
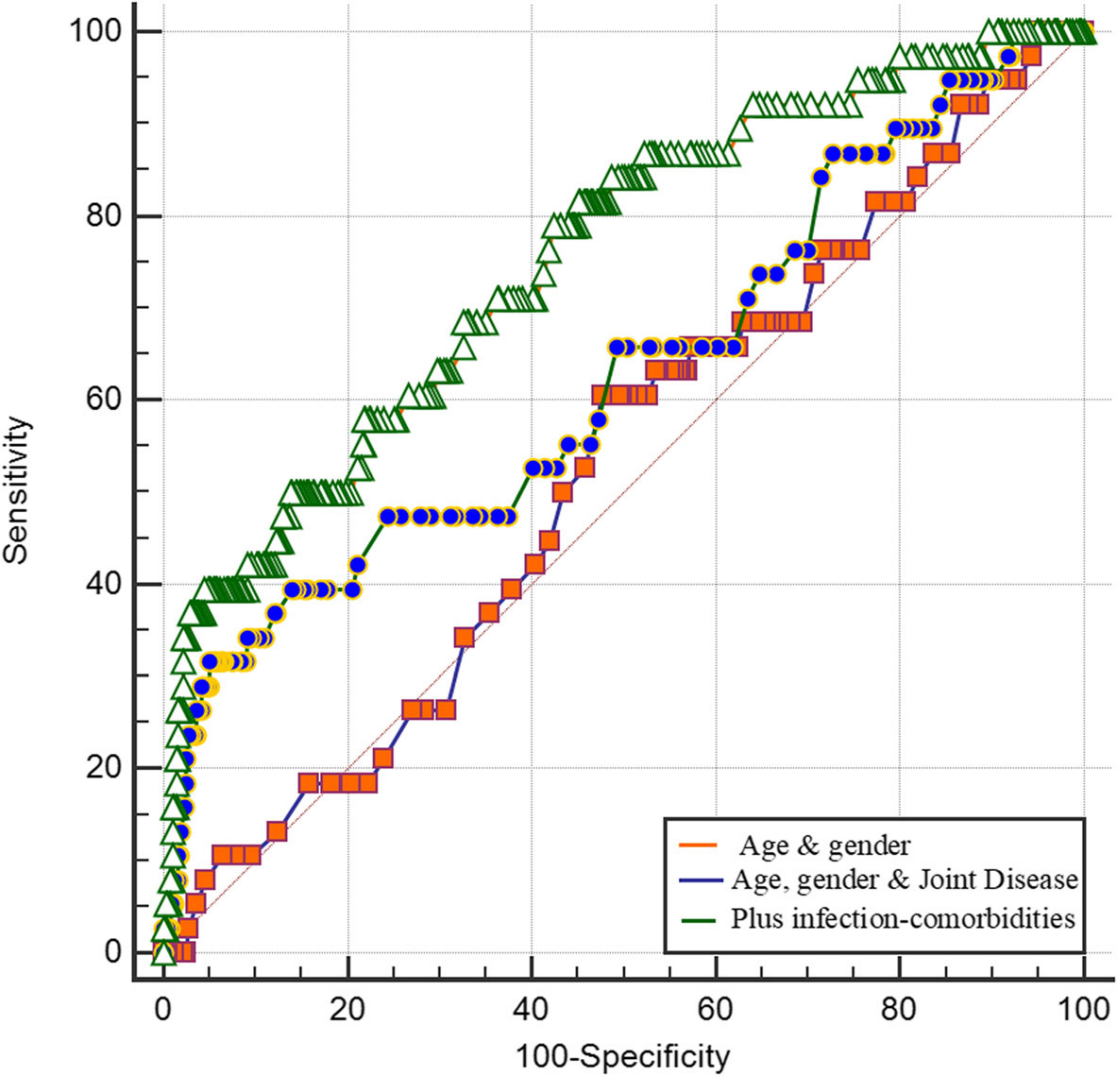
Comparison of areas under the receiver operating characteristic curve (AUROC) for complications due to infection. Fist model (orange line) included only age and gender of patients, second model (blue line): age, gender, and type of joint disease: osteoarthritis or rheumatoid arthritis. Last model (green line); all these factors plus other comorbidities associated with infection. Difference between AUROC significant (*P* < 0.05) for model 3 compared with model 1 and with 2

Risk factors for PJI were RA, use of corticosteroids, and these previous comorbidities: respiratory disease, infections, diabetes, anemia, and mental disease. Most patients with RA had at least another of these infection risk-associated factors; 52% of RA cases having two or more. Odds of having PJI proportionally increased according with the presence of these comorbidities (Fig. 4). Patients with OA and one or two PJI-risk factors had two and half times increased odds for PJI (Fig. 4, OR = 2.5 *P* = 0.05). Patients with OA and three PJI risk factors had five times increased odds of having PJI (Fig. 4, OR = 5.0, *P* = 0.003). Patients with RA had the highest risk for PJI when compared to patients with OA without comorbidities (Fig. 4, OR = 13.6, *P* < 0.001). A model including joint disease (OA or RA) and the number of PJI-associated risk factors (Fig. 5: AUC = 0.76) was superior to predict cases of PJI than a model including type of joint disease (Fig. 5: AUC = 0.63, *P* = 0.0002 for AUROCs comparison) and to a model including only age and gender (Fig. 5, AUC = 0.53, *P* = 0.01, for difference between AUROCs).

## Discussion

The current study revealed that it is possible to identify groups at higher risk for infections and mechanical complications after arthroplasty. There are two groups at higher risk for infections: patients with OA with at least two other risk factors for infections (use of corticosteroids, anemia, previous infections, respiratory disease, diabetes, or mental comorbidity) and patients with RA, who usually present at least one other of these risk factors for infection. We also demonstrated that musculoskeletal comorbidities, principally history of osteoporosis and hernia, are associated with mechanical complications (osteolysis and dislocations, respectively), and they help to predict mechanical complications. Based on these findings, we proposed a simple predictive tool to determine the risk based on the clinical history of the patient (comorbidities) and the type of joint disease (OA/RA). This tool is based on a model where patients with RA will be at higher risk than patients with OA and where the risk will proportionally increase with presence of these factors.

RA was the most significant predictor of all type of complications after TJA. Patients with RA had approximately six and three times higher odds for infections and mechanical complications than subjects with OA. This is the first study to analyze the value of RA and other associated risk factors as predictors of complications after TJA. Previous studies found that patients with RA are at higher risk for PJI and dislocations, without further explanations [7, 12, 13, 15, 17]. According to our results, one of the reasons for subjects with RA of having higher risk for complications due to infections after arthroplasty might be the presence of other comorbidities or corticosteroids use. More than 50% of subjects with RA and PJI or superficial infections after arthroplasty had two other risk factors for infections, which might explain a substantial part of the increased risk for infections after arthroplasty in RA.

The ability to discriminate between patients that might present infections or not after TJA was considered as fair and significant for RA in addition to other infection-related risk factors (respiratory, anemia, history of infections, diabetes, mental diseases, and corticosteroids use) when compared with a model based on the baseline characteristics of the patients and/or type of joint disease (OA/RA). However, not only patients with RA are at higher risk of these type of complication, patients with OA with two or more of these comorbidities are also at higher risk when compared with patients with OA without comorbidity.

Classification and selection of patients (high or low risk for complications) might decrease the number of complications. Patients with a higher complication risk can be more closely monitored/treated to control their risk factors (diabetes, anemia, osteoporosis, respiratory diseases, and other infections) and therefore decrease the chance of complication. Considering the high number of patients in waiting list for surgery in our hospital and many others, it might be possible to select between two similar candidates for surgery based on which has a lower complication risk.

Our study has some strengths and limitations. This is the first study to demonstrate the predictive value of some of the most important patient-related risk factors for infections and mechanical complications after arthroplasty. We also found evidence of the use of corticosteroid and mental comorbidities as risk factors for infection after arthroplasty. These two factors are still controversial or without strong evidence. Only depression has been associated with an increased risk of PJI without further evidence of association of other mental comorbidities [18]. Use of corticosteroids has been mentioned as risk factor for any infection in subjects with RA [7, 19]. Medications used to treat RA have well-defined immunosuppressive effects and infection risk increases with both, doses and duration of corticosteroids therapy [19]. Our results add evidence regarding association of the use of corticosteroids (Prednisone) with deep and superficial infections after arthroplasty. The other risk factors, such as anemia, previous infections, respiratory disease (principally infections), and diabetes, have been widely discussed in the literature as risk factors for PJI. Diabetes and poor preoperative glucose control are not only a risk factor for prosthesis joint infection (PJI) but for deep vein thrombosis, periprosthetic fracture, aseptic loosening, and poorer joint function after surgery as well [12, 14, 20]. Any previous infection is predictor of PJI, especially in subjects that undergo knee arthroplasty [11, 13, 17]. Regarding infections in patients with RA, respiratory tract infections are very common and might be one important source for PJI (hematogenous infection) that has not been studied [21]. In addition, concerning other risk factors for infection in patients with RA, anemia is very prevalent in this population and it has effect on infection risk [17]. Other studies have reported that around 25% of subjects with RA have anemia and it has been advised to evaluate and treat preoperatively this condition to decrease secondary complications risk [22]. In our population, around 15% of subjects with RA had history of anemia/iron deficiency. Despite that hemogram was normal before surgery, history of anemia remained important for infection.

We found some evidence that musculoskeletal comorbidities (history of hernias or osteoporosis) might specifically affect the risk of prosthesis dislocation and loosening of the implant. However, more research is necessary to confirm this association, especially regarding hernia. Osteoporosis has three major potential complications for TJA: perioperative fracture, an increased risk of periprosthetic fracture, and a risk of late aseptic loosening due to mechanical failure of ingrown trabecular bone [23]. In animal studies, bisphosphonates have shown promising results in the prevention and treatment of aseptic loosening and prosthesis osseointegration in THA [24]. More research will be necessary to determine other aspects regarding osteoporosis treatment before TJA, as for example, effect on TKR, number needed to treat to prevent these bone-related complications, among others. This is the first study pointing hernia as a risk factor for prosthesis dislocation. However, there are previous studies linking hernia and hip dislocation. It has been found that children with congenital dislocation of the hip (CDH) sustained inguinal hernia abnormally early in life [25]. The authors of the study suggested that relaxin (which stimulates collagenase) might be responsible for alteration in connective tissue being of importance for the development of both CDH and the hernia. More research is needed to verify this finding and to understand the association between dislocation and hernia.

The main limitation of this study is its retrospective design and small sample size in the RA group. Larger studies are needed to validate our results strengthening the power to confirm some of these risk factors and their value as predictors. It is possible that our study underestimated relatively minor complications such as superficial infections, considering its retrospective design and the fact that we only added minor complications when they were reported by the patient to the hospital or when they were found by the specialist. In addition, despite adjustment for important confounders, there might be other potential confounders that were not included.

## Conclusions

In conclusion, type of joint disease (OA or RA) and history of these comorbidities: musculoskeletal, respiratory diseases, infections, diabetes, anemia, mental disease, and corticosteroids use, might contribute to prediction of mechanical complications and infections before arthroplasty. Patients with OA with at least two other risk factors for infections and patients with RA should be monitored and counseled before surgery regarding their higher risk for PJI. Information about comorbidities and type of joint disease helps to identify patients at higher risk for infection and mechanical complications after TJA.

## Abbreviations

AUC: Areas under the curve
AUROC: Receiver operating characteristic curve
CI 95%: Confidence intervals
HR: Hazard ratios
OA: Osteoarthritis
OR: Odds ratios
PJI: Prosthetic joint infection
RA: Rheumatoid arthritis
TJA: Total joint arthroplasty
